# Subcutaneous encapsulated fat necrosis

**DOI:** 10.1002/ccr3.508

**Published:** 2016-03-10

**Authors:** Dogu Aydin, Jais O. Berg

**Affiliations:** ^1^ Department of Plastic and Reconstructive SurgeryHerlev HospitalUniversity of CopenhagenCopenhagenDenmark

**Keywords:** fat tissue, ischemia, subcutaneous encapsulated fat necrosis, trauma, treatment

## Abstract

We have described subcutaneous encapsulated fat necrosis, which is benign, usually asymptomatic and underreported. Images have only been published on two earlier occasions, in which the necrotic nodules appear “pearly” than the cloudy yellow surface in present case. The presented image may help future surgeons to establish the diagnosis peroperatively.

## Quiz Question: What is this Condition and What is the aetiology?

**Figure 1:**
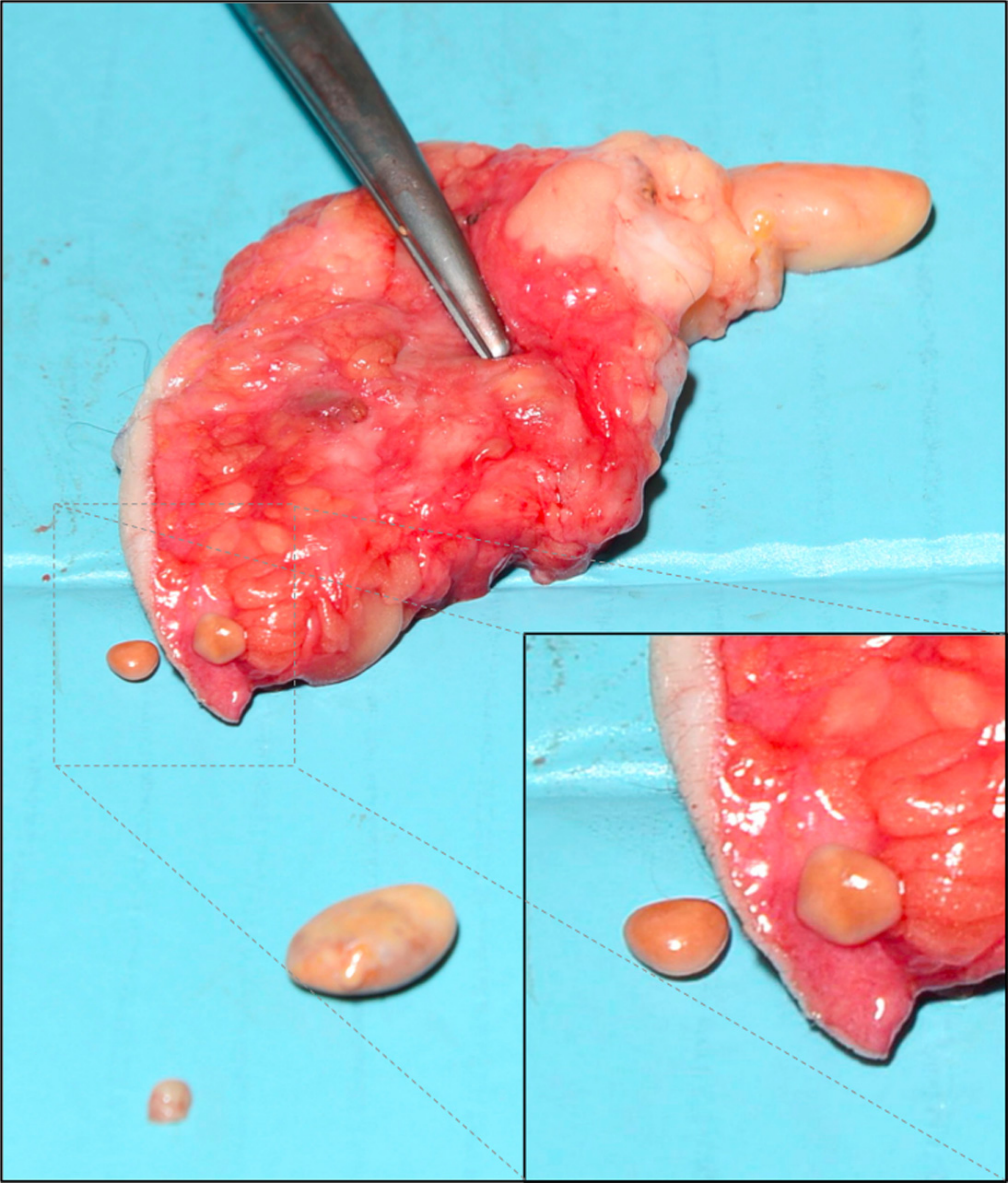
subcutaneous encapsulated fat necrosis.

A tender subcutaneous tumor was excised from the right nates of a 69‐year‐old man. The symptoms subsided after surgery. Pathology revealed encapsulated cystic fat necrosis. This is a benign, usually asymptomatic and underreported condition 1. It is believed to be trauma‐related ischemia and necrosis of fat tissue that gradually detaches from its surroundings. The fibrous capsule hinders reabsorption 1. Only two earlier images have been published, in which the nodules appear “pearly” 2 or white 1 rather than cloudy yellow as the present case. There are no etiological dissimilarities between the two cases and present case. Differences in thicknesses and dystrophic calcifications of the fibrous capsules may explain this variation. Histological examination will exclude malignancy and confirm the diagnosis (Fig. 1).

## Conflict of Interest

None declared.
